# Intraoperative neurophysiological monitoring versus no monitoring in intradural extramedullary tumor surgery: a systematic review and meta-analysis of early postoperative neurological outcomes

**DOI:** 10.1007/s10143-026-04159-3

**Published:** 2026-02-27

**Authors:** Eduardo Mendes Correa, Othavio Lopes, Alexandre Carrion, Domênica Baroni Coelho de Oliveira Ferreira, George Divério, Pedro Henrique Costa Ferreira-Pinto, Flavio Nigri

**Affiliations:** Neurosurgery, Department of Surgical Specialities, Pedro Ernesto University Hospital, State University of Rio De Janeiro, Campus Universitário – Avenida 28 de setembro, 77- Vila Isabel Cep:20, Rio de Janeiro, 551-030 RJ Brazil

**Keywords:** Intradural extramedullary tumors, Intraoperative neurophysiological monitoring, Spinal tumor surgery, Neurological outcomes, Systematic review, Meta-analysis

## Abstract

The role of intraoperative neurophysiological monitoring (IONM) in intradural extramedullary (IDEM) tumor surgery is uncertain. While IONM is standard in intramedullary and deformity procedures, its impact on neurological outcomes in IDEM resections has not been systematically evaluated. To assess whether IONM reduces early postoperative neurological morbidity in IDEM tumor surgery. We conducted a PROSPERO-registered systematic review and meta-analysis (CRD420251119538) in accordance with PRISMA 2020. Comparative studies of IDEM resections with versus without IONM were included. Outcomes were harmonized into a binary “new neurological deficit” at discharge. Risk of bias was assessed with ROBINS-I, and certainty of evidence with GRADE. Pooled odds ratios (ORs) were calculated using a random-effects model. Four retrospective cohorts comprising 602 patients (306 with IONM, 296 without) were analyzed. In absolute terms, early postoperative neurological deficits occurred in 19/306 patients (6.2%) in the IONM group versus 45/296 patients (15.2%) in the non-IONM group. Pooled analysis showed a significant reduction in early postoperative neurological deficits with IONM compared to no IONM group (OR 0.39, 95% CI 0.20–0.75; *p* = 0.005; I² = 22%), corresponding to an absolute risk reduction of 9.2% (NNT ≈ 11). Sensitivity analysis revealed dependence on certain large studies. Risk of bias was moderate-to-serious, and certainty of evidence was rated low. IONM use was associated with lower odds of early postoperative neurological deficits in IDEM tumor surgery, and the effect is based on low-certainty observational evidence. These findings are hypothesis-generating and support selective rather than universal use of IONM, particularly in higher-risk cases. Prospective multicenter studies with standardized outcomes, longer follow-up, and cost-effectiveness analyses are needed to define its optimal role.

**Supplementary Information:**

The online version contains supplementary material available at 10.1007/s10143-026-04159-3.

## Introduction

Intradural extramedullary (IDEM) tumors constitute a significant proportion of adult and pediatric spinal neoplasms, with meningiomas and nerve sheath tumors being the most common histological subtypes [5]. Surgical resection remains the mainstay of treatment and is often curative. Despite being located outside the spinal cord parenchyma, IDEM tumors are frequently adherent to neural structures, particularly in long-standing lesions, increasing the risk of iatrogenic neurological deficits following resection [9, 16]. Given the functional consequences associated with motor or sensory injury, ensuring neurological preservation during surgery is paramount.

Intraoperative neurophysiological monitoring (IONM), particularly the use of somatosensory evoked potentials (SSEPs) and motor evoked potentials (MEPs), has become standard practice in surgeries involving intramedullary spinal cord tumors and deformity correction [15]. However, its role in IDEM tumor resection remains less clearly defined [10, 14]. While some centers routinely employ IONM in all spinal tumor cases, others reserve it for high-risk locations or complex cases. This variability reflects an ongoing uncertainty regarding the true impact of IONM in extramedullary lesions, where the expected neurological risk is often perceived to be lower than in intramedullary procedures.

Although multiple systematic reviews and meta-analyses have evaluated IONM in spinal surgery, these studies have primarily focused on diagnostic accuracy, assessing the ability of somatosensory and motor evoked potentials to predict postoperative neurological injury. Importantly, diagnostic performance does not necessarily translate into improved patient outcomes.

In contrast, the present study focuses specifically on comparative clinical outcomes, synthesizing available evidence to evaluate whether the use of IONM during resection of IDEM tumors is associated with a reduction in early postoperative neurological morbidity compared with surgery performed without monitoring. By restricting inclusion to IDEM tumors and harmonizing heterogeneous outcome reporting into a standardized early neurological endpoint, this meta-analysis addresses a clinically relevant question that has not been previously examined quantitatively.

## Methods

This systematic review and meta-analysis was conducted in accordance with the Preferred Reporting Items for Systematic Reviews and Meta-Analyses (PRISMA 2020) guidelines. The study protocol was defined a priori and registered in the International Prospective Register of Systematic Reviews (PROSPERO; registration ID: CRD420251119538; https://www.crd.york.ac.uk/PROSPERO/view/CRD420251119538*)*.

We included comparative studies that evaluated surgical outcomes in patients undergoing resection of histologically confirmed IDEM spinal tumors, with or without the use of IONM. Eligible studies were required to report data on postoperative neurological status in both monitored and non-monitored groups. Any study design (retrospective or prospective) was eligible, provided that a comparator group was available. Studies focusing exclusively on intramedullary tumors, those lacking extractable postoperative outcome data, or those in which all patients received IONM were excluded. Case reports, non-comparative case series, conference abstracts without full text, and duplicate publications were also excluded. To maximize sensitivity, histology-specific terms were not included in the search strategy. Only studies published in English were considered eligible for inclusion.

### Search strategy

A comprehensive search strategy was developed and applied to three electronic databases: PubMed, Embase, and Web of Science. The search was conducted on July 20, 2025, in accordance with PRISMA 2020 guidelines. We used a combination of Medical Subject Headings (MeSH), Emtree terms, and free-text keywords to identify studies related to spinal tumors, intraoperative neurophysiological monitoring, and postoperative neurological outcomes. Searches were conducted exclusively in bibliographic databases; no clinical trial registries or study registers were queried.

To ensure full reproducibility and transparency, the complete database-specific search strategies are reported in detail below.

1. PubMed: (“Spinal Cord Neoplasms“[MeSH] OR “Spinal Neoplasms“[MeSH] OR spinal tumor*[tiab] OR spinal cord tumor*[tiab] OR spinal neoplasm*[tiab] OR intramedullary tumor*[tiab] OR extramedullary tumor*[tiab]) AND (“Intraoperative Neurophysiological Monitoring“[MeSH] OR intraoperative monitoring[tiab] OR neurophysiological monitoring[tiab] OR IONM[tiab] OR MEP[tiab] OR SSEP[tiab] OR “motor evoked potential*“[tiab] OR “somatosensory evoked potential*“[tiab] OR D-wave[tiab] OR electromyography[tiab] OR EMG[tiab]) AND (outcome*[tiab] OR complication*[tiab] OR neurologic*[tiab] OR deficit*[tiab] OR reoperation[tiab] OR “treatment outcome“[MeSH] OR “postoperative complications“[MeSH])
2. Embase: (‘spinal cord neoplasm’/exp OR ‘spinal neoplasm’/exp OR ‘spinal tumor’:ti, ab OR ‘spinal cord tumor’:ti, ab OR ‘spinal neoplasm’:ti, ab OR ‘intramedullary tumor’:ti, ab OR ‘extramedullary tumor’:ti, ab) AND (‘intraoperative neurophysiological monitoring’/exp OR ‘intraoperative monitoring’:ti, ab OR ‘neuromonitoring’:ti, ab OR ‘ionm’:ti, ab OR ‘meps’:ti, ab OR ‘motor evoked potential’:ti, ab OR ‘sseps’:ti, ab OR ‘somatosensory evoked potential’:ti, ab OR ‘d wave’:ti, ab OR ‘electromyography’:ti, ab OR ‘emg’:ti, ab) AND (‘treatment outcome’/exp OR outcome*:ti, ab OR complication*:ti, ab OR neurologic*:ti, ab OR deficit*:ti, ab OR reoperation*:ti, ab OR ‘postoperative complication’/exp).
3. Web Of Science (WOS): TS = ((“spinal tumor*” OR “spinal cord tumor*” OR “spinal neoplasm*” OR “intramedullary tumor*” OR “extramedullary tumor*”) AND (“intraoperative monitoring” OR “neurophysiological monitoring” OR “IONM” OR “MEP” OR “SSEP” OR “D-wave” OR “motor evoked potential*” OR “somatosensory evoked potential*” OR “EMG”) AND (outcome* OR complication* OR neurologic* OR deficit* OR reoperation*)).

All records were imported into the Rayyan platform for duplicate removal and blinded screening. Two reviewers independently screened titles and abstracts, followed by full-text evaluation. Disagreements were resolved through consultation with the senior author. Data extraction was performed using a standardized form and included study design, patient demographics, tumor histology, location, IONM modalities, outcome definitions, and follow-up duration [12]. For histological classification, nerve sheath tumors (NST) were treated as a single category. When studies reported schwannomas specifically, these cases were grouped under NST, as schwannomas represent a subset of nerve sheath tumors, to ensure consistency across heterogeneous reporting. We assessed potential overlap of patient cohorts by reviewing study institutions, recruitment periods, and found no evidence of overlapping populations among the included studies. Reference lists of included studies were manually screened to identify additional eligible articles. Disagreements during title/abstract screening, full-text review, and data extraction were resolved by consensus between the two reviewers; no third-reviewer adjudication was required, and interrater agreement statistics were not formally calculated.

### Outcome definition and harmonization

The primary outcome was defined a priori as the presence of a new or worsened neurological deficit at hospital discharge or within 30 postoperative days (early deficit). When multiple postoperative timepoints were available, the earliest assessment within this timeframe was used for the primary analysis, in order to standardize outcome ascertainment across studies.

Study-specific definitions of postoperative neurological deficit, neurological domains assessed, and outcome timepoints, as well as the corresponding mapping decisions applied in this meta-analysis, are detailed in Supplementary Table 1. When studies reported neurological outcomes at multiple postoperative timepoints, only the earliest assessment within the predefined early postoperative window was used for quantitative synthesis, in accordance with the prespecified primary outcome. Because postoperative neurological outcomes were reported heterogeneously across studies, a prespecified harmonization strategy was applied to enable valid quantitative synthesis. Postoperative neurological status was transformed into a binary variable (deficit present vs. absent) using a hierarchical mapping approach, as follows:

1. Deterioration by at least one grade on a validated functional scale (e.g., Modified McCormick, Frankel).
2. Explicit report of new motor, sensory, or sphincter dysfunction not present preoperatively.
3. Classification as “neurological deterioration” or “postoperative neurological deficit” as defined by the original study authors.

When multiple neurological domains were reported, the occurrence of any deficit was classified as an event. Differences in outcome definitions and assessment timepoints were recorded at the study level and are summarized below. Residual heterogeneity related to outcome definition and timing is acknowledged as an inherent limitation of the available observational evidence.

Per-study outcome mapping and timepoint selection were as follows:

- Cofano et al. (2020) [4]: Modified McCormick Scale at discharge; deterioration at discharge classified as a deficit.
- Mirza et al. (2024) [11]: Global motor assessment; patients rated as “deterioration” at discharge classified as having a deficit.
- Cabañes-Martínez et al. (2024) [2]: Direct extraction of the “postoperative neurological deficit” variable as defined and reported by the study authors.
- Harel et al. (2017) [7]: “new neurological deterioration” variable; new neurological deterioration classified as having a deficit.

### Bias assessment

Risk of bias for non-randomized studies was assessed independently by two reviewers using the ROBINS-I tool, which classifies each of seven domains as Low, Moderate, Serious, or Critical risk of bias. Domains included: (1) bias due to confounding; (2) bias in selection of participants; (3) bias in classification of interventions; (4) bias due to deviations from intended interventions; (5) bias due to missing data; (6) bias in measurement of outcomes; and (7) bias in selection of the reported result [17]. The certainty of evidence for the primary outcome was assessed using the GRADE approach. Because all included studies were non-randomized observational cohorts, the certainty of evidence started at low and was subsequently rated down or up according to standard GRADE domains, including risk of bias, inconsistency, indirectness, imprecision, and publication bias. Any considerations for upgrading (e.g., large magnitude of effect or residual confounding that would reduce the observed association) were applied cautiously and only when supported by the data [6]. Domain-level justifications for ROBINS-I judgments for each included study are provided in Supplementary Table 2.

## Statistical analysis

Meta-analyses were performed using unadjusted arm-level event data, as adjusted effect estimates were not consistently available across studies. A random-effects model (Mantel–Haenszel method) was used to generate pooled odds ratios (ORs) and 95% confidence intervals (CIs), reflecting expected clinical and methodological heterogeneity across observational cohorts. Heterogeneity was assessed using the I² statistic.

With only four included studies, random-effects meta-analysis is inherently unstable and should be interpreted cautiously. Alternative approaches such as Hartung–Knapp adjustment or prediction intervals were considered; however, given the very small number of studies and heterogeneous designs, we prioritized transparent reporting of sensitivity analyses and explicitly framed the findings as exploratory.

Subgroup analyses were prespecified (spinal level, histology, monitoring modality, and baseline neurological status). However, due to insufficient and non-harmonized stratum-level reporting across studies (≤ 2 studies per stratum; heterogeneous definitions and timepoints), no quantitative subgroup meta-analyses or tests for interaction were feasible. Therefore, the inability to perform quantitative subgroup analyses represents an important limitation of the available evidence and should be considered when interpreting the pooled results. Quantitative synthesis was therefore restricted to the primary outcome (new or worsened neurological deficit at discharge or ≤ 30 days). Sensitivity analyses were limited to leave-one-out. All statistical analyses were conducted using Review Manager (RevMan 5.4).

Secondary outcomes of interest included extent of resection (EOR; gross-total vs. subtotal, as defined by each study), length of stay (LOS), and postoperative complications (e.g., CSF leak/pseudomeningocele, wound infection, reoperation, recurrence). Owing to heterogeneous definitions, incomplete per-arm reporting, and non-comparable summary statistics across studies, no quantitative meta-analyses were performed for these outcomes. Instead, we provide a structured narrative synthesis.

To improve clinical interpretability, relative effect estimates were translated into absolute risk differences using the median baseline risk observed in the control groups. Absolute risks were calculated by converting odds ratios to risk estimates according to the following formula:

*Risk_IONM = OR × Risk_control/[1 − Risk_control + (OR × Risk_control)]*.

Absolute risk difference was then derived as the difference between control and IONM risks, and the number needed to treat (NNT) was calculated as the inverse of the absolute risk difference. These estimates are presented as illustrative translations of relative effects rather than definitive measures of treatment benefit.

## Results

**Fig. 1:**
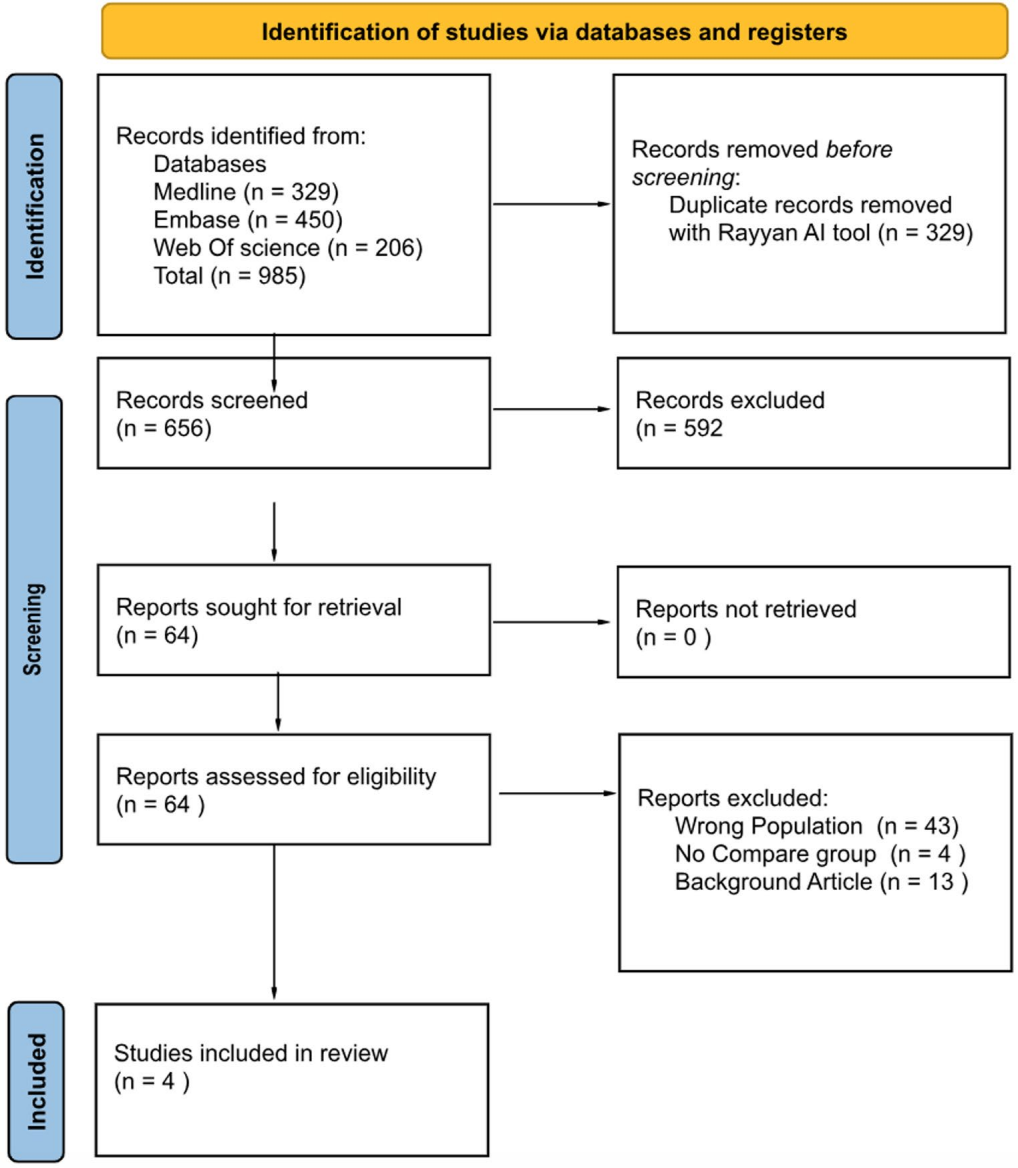
PRISMA 2020 flow diagram of study selection for the systematic review and meta-analysis. The diagram summarizes the number of records identified, screened, assessed for eligibility, and included, with reasons for exclusion at each stage

The initial search yielded 985 records across all databases: 329 from PubMed, 450 from Embase, and 206 from Web of Science. After removal of 329 duplicates, 656 titles and abstracts were screened. A total of 64 full-text articles were assessed for eligibility. Of these, 60 were excluded: 43 for wrong population, 13 for being background or review articles, and 4 for lacking a comparator group. A total of four studies evaluating intradural extramedullary (IDEM) spinal tumors were included in the meta-analysis. The study selection process is summarized in Fig. 1.

The included studies were retrospective cohort analyses published between 2017 and 2024, encompassing a total of 602 patients who underwent surgical resection of intradural extramedullary spinal tumors. Among these, 306 patients underwent surgery with intraoperative neurophysiological monitoring (IONM), while 296 were operated on without IONM. Female patients range from 43% to 67.5%. The mean age across studies varied from 50.7 to 60.5 years, with comparable distributions between the IONM and non-IONM groups.

Tumor location was heterogeneous, with thoracic lesions being the most common, reported in 42.3% to 60% of patients. Cervical tumors ranged from 14% to 21%, lumbar tumors from 9% to 38.5%, and cervicothoracic/thoracolumbar transition lesions in 4.3% to 10% of cases. Histologically, the most frequently reported tumor types were meningiomas (32.5% to 68.4%) and nerve sheath tumors, including schwannomas (30% to 43.7%). Ependymomas ranged from 10% to 14% and other rare histologies accounted for zero up to 21.5% of the tumors in some studies.

All studies utilized multimodal IONM, most commonly combining somatosensory evoked potentials (SSEPs) and motor evoked potentials (MEPs). Two studies also included D-wave monitoring, and two incorporated electromyography (EMG). The follow-up duration varied across studies, ranging from 3 months to 48.3 months. Detailed characteristics of the included studies are presented in Table 1 The primary outcome assessed was the incidence of new postoperative neurological deficits (NND) in patients who underwent surgery with IONM compared to those without IONM

**Table 1:** Summarizes the characteristics of the included studies : study design, sex, age, sample size by group, tumor location, histology, IONM modalities used, and follow-up duration

Study	Study Design	Sex (%)	Age (Y) summary as reported)	Sample Size (IONM/No IONM)	Tumor Location (%)	Histology (%)	IONM Modalities	FU
Cofano et al. (2020) [4]	Retrospective cohort	F = 64.7	58 (range 18–88)	162/87	Cervical = 17.3 thoracic = 43.8 lumbar = 38.5	Meningioma = 37.7 NST = 43.7 Ependymoma = 12 Others = 6.6	MEP, SSEP, D-Wave	48.3Mo
Harel et al. (2017) [7]	Retrospective cohort	F = 67.5	56 60.5	41/70	Cervical = 21 Thoracic = 60 Lumbar = 9 Transition = 10	Meningioma = 68.4 NST = 31.6	SSEP, MEP, EMG	NR
Cabañes-Martinez et al. (2024) [2]	Retrospective cohort	F = 57	50.66 ± 17.48 54.49 ± 17.06	32/47	Cervical = 14 Thoracic = 47 Lumbar = 29 Transition = 10	Meningioma = 32.5 NST = 38 Ependymoma = 10 Others = 19.5	SSEP, MEP, D-wave, EMG	3Mo
Mirza et al. (2024) [11]	Retrospective cohort	F = 43	55.69 ± 1.83 55.16 ± 1.81	71/92	Cervical = 18.4 Thoracic = 42.3 Lumbar = 34.3 Transition = 4.3	Meningioma = 35.5 NST = 30 Ependymoma = 14 Others = 21.5	MEP SSEP	6Mo

IDEM: intradural extramedullary; IONM: intraoperative neurophysiological monitoring; MEP: motor evoked potentials; SSEP: somatosensory evoked potentials; EMG: electromyography; D-wave: direct corticospinal tract (descending) D-wave recording; NST: nerve sheath tumor; EOR: extent of resection; FU: follow-up; NR: not reported; SD: standard deviation; IQR: interquartile range; Y: years; Mo: months; F/M: female/male; CTJ: cervicothoracic junction; TLJ: thoracolumbar junction

Age values are mean ± SD unless otherwise specified. “Transition” refers to junctional levels (CTJ or TLJ). Footnote NST: nerve sheath tumors. When studies reported schwannomas separately, these were classified under the NST category, as schwannomas represent a subset of nerve sheath tumors. Footnote: Age is reported using the summary statistics provided by each study. When stratification by IONM status was not reported, a single cohort-level value is shown. Measures of dispersion (SD, SE, or range) are presented as reported by the original authors, without conversion or imputation

Secondary outcomes included extent of resection, length of stay, and postoperative complications. Across studies, these outcomes were frequently reported only at the cohort level or without stratification by monitoring status, precluding numerical comparison between IONM and non-IONM groups. A structured per-study summary, indicating which secondary outcomes were numerically extractable and which were not stratified by the original authors, is provided in Supplementary Table 3.

### Main analysis

**Fig. 2:**
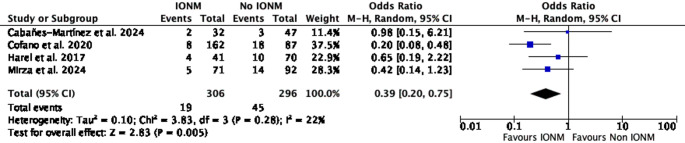
Forest plot of the meta-analysis comparing the incidence of new postoperative neurological deficits in patients undergoing resection of intradural extramedullary spinal tumors with intraoperative neurophysiological monitoring (IONM) versus without IONM. IONM use was associated with lower odds of early postoperative neurological deficits

The pooled analysis demonstrated that IONM was associated with a statistically significant reduction in the odds of developing new neurological deficits in patients with IDEM tumors. The random-effects model yielded an odds ratio (OR) of 0.39 (95% confidence interval [CI], 0.20 to 0.75; *p* = 0.005), with heterogeneity I² = 22% (Fig. 2). No subgroup meta-analyses were performed because stratum-level data were insufficient and non-harmonized across studies; quantitative pooling was therefore restricted to the early postoperative neurological deficit. Using the median control risk, the pooled estimate corresponds to an absolute risk reduction of 9.2% for early neurological deficit with IONM (Summary of Findings, Table 2), yielding an approximate NNT ≈ 11 to prevent one early postoperative neurological deficit.

**Table 2:** Summary of findings (SoF) Table – GRADE approach

Outcome	Relative Effect (OR [95% CI])	Absolute Effect (Risk Difference)*	Certainty of Evidence (GRADE)	Interpretation
New neurological deficit (IDEM tumors)	0.39 [0.20–0.75]	−9.2% fewer events with IONM	⬤⬤◯◯ Low	IONM use is associated with fewer early postoperative neurological deficits.
Sensitivity analysis (Leave-one-out)	0.34–0.56 [0.15–1.18]	Effect direction preserved; statistical significance lost with exclusion of influential studies	—	Effect trend robust to study removal.

* Absolute risk reduction estimated using the median baseline risk in the control group

Downgraded one level for risk of bias: all included studies were observational, starting at Low certainty, and one study was rated at ***Serious*** risk for confounding due to lack of adjustment for prognostic factors (baseline neurological status, tumor location)

Downgraded one level for inconsistency: heterogeneity in outcome definitions, follow-up timepoints, and sensitivity analyses showing that exclusion of certain influential studies (Cofano, Mirza) eliminated statistical significance

No upgrading: although the point estimate (OR 0.39) meets the threshold for a moderate protective effect (≤ 0.5), the confidence interval extends above 0.5 and residual confounding could plausibly attenuate the observed association

The certainty of evidence reflects observational data and should be interpreted as indicating the level of confidence in an association rather than a causal effect

Absolute risk reduction and number needed to treat were calculated using the median control-group risk and should be interpreted as illustrative translations of the relative effect estimate, not as definitive or causal measures

### Sensitivity Analysis – Leave-One-Out

**Fig. 3:**
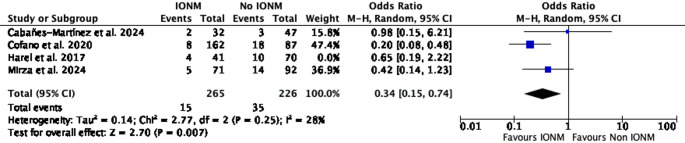
Leave-one-out sensitivity analysis excluding Harel et al. The pooled estimate remained statistically significant, favoring IONM use

**Fig. 4:**
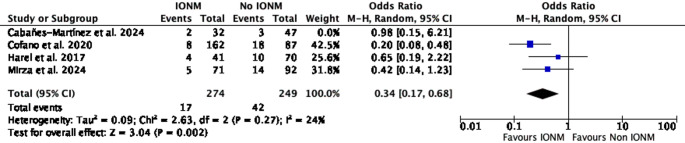
Leave-one-out sensitivity analysis excluding Cabañes-Martínez et al. The pooled estimate remained statistically significant, favoring IONM use

**Fig. 5:**
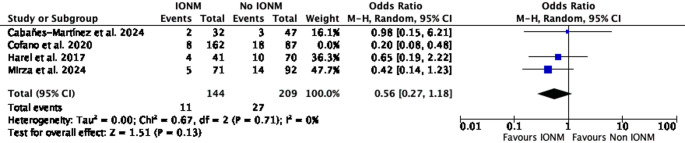
Leave-one-out sensitivity analysis excluding Cofano et al. The pooled estimate was no longer statistically significant, indicating sensitivity of the overall effect to exclusion of this study

**Fig. 6:**
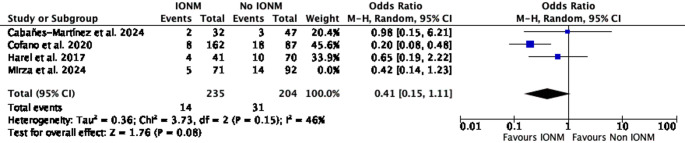
Leave-one-out sensitivity analysis excluding Mirza et al. The pooled estimate was no longer statistically significant, indicating sensitivity of the overall effect to exclusion of this study

A leave-one-out sensitivity analysis was conducted to assess the influence of individual studies on the pooled estimate of effect. When Harel et al. was excluded, the pooled effect remained statistically significant (OR = 0.34; 95% CI 0.15–0.74; *p* = 0.007; I² = 28%). Similarly, exclusion of Cabañes-Martínez et al. retained statistical significance (OR = 0.36; 95% CI 0.17–0.68; *p* = 0.002; I² = 24%) (Figs. 3 and 4). In contrast, exclusion of Cofano et al. resulted in loss of statistical significance (OR = 0.56; 95% CI 0.27–1.18; *p* = 0.13; I² = 0%). Likewise, omission of Mirza et al. yielded a non-significant pooled estimate (OR = 0.41; 95% CI 0.15–1.11; *p* = 0.08; I² = 46%) (Figs. 5 and 6).

Risk of bias was assessed using the ROBINS-I tool for non-randomized studies. Among the included studies, all were judged to have a moderate to serious risk of bias, primarily due to concerns related to confounding and selection of participants. No study was classified as having a critical risk of bias, and outcome measurement was generally considered appropriate across studies (Table 3). For the primary outcome of new neurological deficit at discharge or ≤ 30 days, the certainty of evidence was rated as LOW. This rating reflects: (1) downgrading for risk of bias due to the observational design of all included studies and the presence of at least one study with *Serious* risk for confounding; (2) downgrading for inconsistency, as the pooled effect was sensitive to the removal of certain large studies in leave-one-out analysis; and (3) no upgrading, as although the pooled effect size was clinically meaningful (OR ~ 0.39), the confidence intervals did not meet the GRADE “large effect” criteria and residual confounding could plausibly attenuate the association. Evidence for late postoperative deficits was rated as very low due to sparse and heterogeneous reporting (Table 2).

**Table 3:**
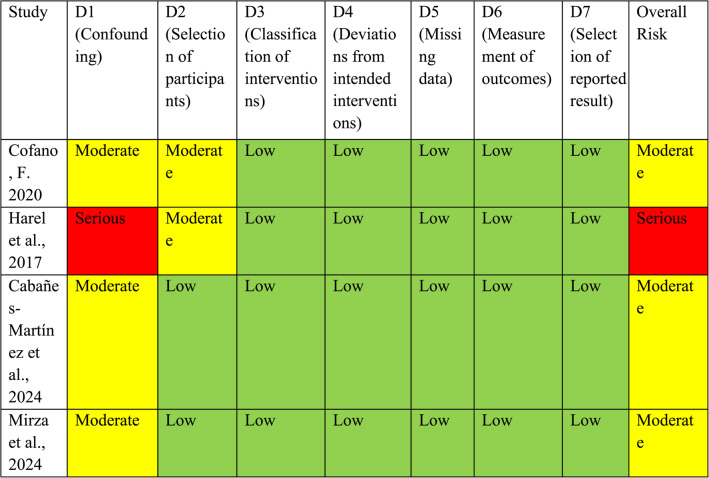
Risk of Bias Assessment Using ROBINS-I Tool for Included Studies Comparing IONM vs. No IONM in Spinal Tumor Surgery

D1. Confounding: Bias due to uncontrolled differences in prognostic factors

D2. Selection of participants: Bias if participants included are not representative or selected non-consecutively

D3. Classification of interventions: Whether IONM vs. no IONM was clearly defined and pre-specified

D4. Deviations from intended interventions: Bias from deviations after intervention allocation

D5. Missing data: Incomplete follow-up or selective attrition

D6. Measurement of outcomes: Blinding, objectivity, or standardization of outcome measurement

D7. Selection of reported result: Selective reporting of outcomes or timepoints. Color Coding: Low Risk  Moderate Risk High Risk

## Discussion

This meta-analysis demonstrates that the use of intraoperative neurophysiological monitoring (IONM) during resection of intradural extramedullary (IDEM) tumors is associated with a lower risk of early postoperative neurological deficits. Using a random-effects Mantel–Haenszel model, the pooled effect favored IONM, corresponding to an absolute risk reduction of approximately 9.2% based on the median control risk (Summary of Findings—Table 2). Importantly, this study advances the existing literature by moving beyond diagnostic accuracy and directly synthesizing comparative clinical outcome data from IDEM tumor surgeries performed with and without IONM.

Previous systematic reviews and meta-analyses in spinal surgery have primarily focused on the predictive performance of IONM signals—demonstrating high sensitivity and specificity of somatosensory evoked potentials (SSEP), motor evoked potentials (MEP), or multimodal monitoring for detecting impending neurological injury [1, 8, 13, 18]. However, predictive accuracy does not necessarily translate into improved patient outcomes. To our knowledge, no prior meta-analysis has specifically evaluated whether IONM use is associated with fewer postoperative neurological deficits compared with unmonitored IDEM resections. By restricting inclusion to IDEM tumors and harmonizing heterogeneous outcome reporting into a standardized early neurological endpoint, the present study addresses this clinically relevant and previously unquantified question.

In leave-one-out sensitivity analyses, the pooled association was sensitive to the exclusion of individual large studies. Omission of Cofano et al. or Mirza et al. resulted in loss of statistical significance (Figs. 5 and 6) [4, 11], whereas exclusion of Harel et al. or Cabañes-Martínez et al. had minimal impact on the summary estimate [2, 7]. This pattern indicates that the observed association is influenced by a limited number of cohorts, highlighting the restricted robustness of the current evidence base and reinforcing the need for cautious interpretation. Importantly, because intraoperative neurophysiological monitoring comprised different modality combinations and alert criteria across studies, it was analyzed as a composite intervention, precluding attribution of the observed association to any single monitoring technique.

Interestingly, studies included in this meta-analysis that reported the strongest early neurological benefit associated with IONM use did not demonstrate a corresponding difference in extent of resection (EOR) when compared with unmonitored cases, as reported by Cofano et al. and Cabañes-Martínez et al. This apparent dissociation between neurological outcomes and EOR gives rise to two non-mutually exclusive, speculative hypotheses. The “protective restraint” hypothesis suggests that intraoperative alerts may prompt surgeons to intentionally limit tumor resection in order to preserve neurological function, thereby preventing deficits without necessarily improving oncological radicality. Conversely, the “confident completion” hypothesis proposes that stable neuromonitoring signals may provide reassurance, allowing surgeons to pursue more complete resections without increasing neurological morbidity. The absence of a measurable difference in EOR between monitored and unmonitored groups suggests that these opposing mechanisms may coexist and counterbalance each other at the cohort level. Which dynamic predominates likely depends on tumor location, baseline neurological status, and surgical complexity. These hypotheses should be regarded as exploratory and highlight the need for future prospective studies integrating standardized neurological outcomes with granular measures of surgical radicality and long-term tumor control.

From a clinical perspective, these findings highlight the importance of patient selection. High-risk cases—such as those with cervical tumors, severe preoperative deficits, or recurrent lesions—are the most likely to benefit meaningfully from IONM. In lower-risk cases, (e.g. posterior, thoracic small tumors) where the likelihood of new deficits is low and transient deficits tend to resolve, the incremental value of IONM may be limited. Although IDEM-specific economic evaluations are scarce, cost-effectiveness analyses in broader spinal surgery contexts suggest that IONM may be cost-saving when morbidity rates exceed 0.3%, with estimated lifetime savings exceeding USD 23,000 per avoided deficit [3]. Selective rather than universal deployment may therefore optimize both clinical and economic outcomes.

Several limitations must be acknowledged. All included studies were retrospective observational cohorts, and pooled estimates were derived from unadjusted arm-level data, limiting causal inference and leaving the results susceptible to residual confounding. Key prognostic factors—such as tumor location, baseline neurological status, lesion size, surgical complexity, and surgeon or center experience—were not consistently adjusted for and may have influenced both IONM utilization and outcomes. Although a prespecified harmonization strategy enabled quantitative synthesis, postoperative neurological outcomes were reported heterogeneously with respect to definitions, domains, and assessment timepoints. Follow-up was generally short and inconsistently reported, restricting interpretation to early postoperative outcomes and precluding conclusions regarding long-term neurological recovery, recurrence, or durability of benefit. Finally, the small number of eligible comparative studies and lack of harmonized stratum-level reporting precluded quantitative subgroup analyses, limiting generalizability.

Taken together, these observational data suggest an association between IONM use and reduced early postoperative neurological morbidity in IDEM tumor surgery. However, given the low certainty of evidence, residual confounding, and sensitivity to influential studies, these findings should be interpreted as hypothesis-generating rather than confirmatory, supporting selective—rather than universal—use of IONM pending prospective validation.

## Conclusion

Intraoperative neurophysiological monitoring during resection of intradural extramedullary spinal tumors may be associated with a lower risk of early postoperative neurological deficits. However, this association is derived from low-certainty observational evidence and remains sensitive to study-level influence. As such, the findings should be interpreted as hypothesis-generating rather than definitive. Selective use of intraoperative monitoring may be reasonable in higher-risk cases, but well-designed prospective multicenter studies with standardized outcome definitions, longer follow-up, and cost-effectiveness analyses are required to define its optimal role.

## Supplementary information

Below is the link to the electronic supplementary material.

Supplementary file1 (docx 1.61 MB)

## Data Availability

No datasets were generated or analysed during the current study.

## Abbreviations

ARD: Absolute Risk Difference
CI: Confidence Interval
EOR: Extent of Resection
GRADE: Grading of Recommendations Assessment, Development and Evaluation
IDEM: Intradural Extramedullary
IONM: Intraoperative Neurophysiological Monitoring
LOS: Length of Stay
MEP: Motor Evoked Potential
NNT: Number Needed to Treat
NND: New Neurological Deficit
OR: Odds Ratio
PRISMA: Preferred Reporting Items for Systematic Reviews and Meta–Analyses
ROBINS: I–Risk Of Bias In Non–randomized Studies of Interventions
SSEP: Somatosensory Evoked Potential
