# Supplementary material for: Intraoperative neurophysiological monitoring versus no monitoring in intradural extramedullary tumor surgery: a systematic review and meta-analysis of early postoperative neurological outcomes
Source: Neurosurg Rev. 2026 Feb 27;49(1):251. doi: 10.1007/s10143-026-04159-3 (PMC12945926; doi:10.1007/s10143-026-04159-3)
Supplement: Supplementary file 1 — Supplementary file1 (docx 1.61 MB) [file 10143_2026_4159_MOESM1_ESM.docx]

### **Supplementary Table 1 . Per-study definition and timing of postoperative neurological outcomes**

| **Study** | **Outcome definition** | **Domains** | **Time Points reported** | **Other timepoints reported (not analyzed)** | **Time Point used** | **Mapping decision** |
| --- | --- | --- | --- | --- | --- | --- |
| **Cofano et al., 2020** | Modified McCormick Scale; deterioration ≥1 grade | Global | Discharge, last follow-up | Last follow-up neurological status | Discharge | Deterioration at discharge = deficit |
| **Mirza et al., 2024** | Motor status: improved / unchanged / deteriorated | Motor | Discharge, 3 months | 3-month motor outcome | Discharge | “Deterioration” at discharge = deficit |
| **Cabañes-Martínez et al., 2024** | Author-defined postoperative neurological deficit | Motor, sensory, sphincter | Discharge | None reported | Discharge | Direct extraction |
| **Harel et al., 2017** | Frankel + clinical deterioration | Motor ± sensory | Early postoperative, follow-up | Follow-up neurological status | Earliest | New deterioration = deficit |

## **Supplementary Table 2. Domain-level ROBINS-I risk of bias assessment and justification**

| **Study** | **D1 – Confounding** | **D2 – Selection of participants** | **D3 – Classification of interventions** | **D4 – Deviations from intended interventions** | **D5 – Missing data** | **D6 – Measurement of outcomes** | **D7 – Selection of reported result** | **Overall risk** |
| --- | --- | --- | --- | --- | --- | --- | --- | --- |
| **Cofano et al., 2020** | Moderate – Non-random IONM allocation; no adjustment for baseline severity. | Moderate – Retrospective cohort; potential selection bias. | Low – Clear definition of IONM vs no IONM. | Low – No relevant deviations reported. | Low – Complete outcome data. | Low – Standardized neurological assessment at discharge. | Low – No evidence of selective reporting. | Moderate |
| **Harel et al., 2017** | Serious – Strong confounding by case complexity and baseline deficits; no adjustment. | Moderate – Retrospective design; clinical selection. | Low – Monitoring status clearly defined. | Low – No deviations reported. | Low – Outcomes largely complete. | Low – Frankel scale and explicit neurological deterioration. | Low – No selective reporting detected. | Serious |
| **Cabañes-Martínez et al., 2024** | Moderate – Non-random allocation; limited adjustment. | Low – Consecutive inclusion. | Low – Clear IONM description. | Low – No deviations reported. | Low – Complete data. | Low – Explicit postoperative deficit definition. | Low – Outcomes aligned with objectives. | Moderate |
| **Mirza et al., 2024** | Moderate – Surgeon discretion; no multivariable adjustment. | Low – Clear cohort definition. | Low – Groups clearly distinguished. | Low – No deviations. | Low – Complete outcome reporting. | Low – Systematic neurological assessment. | Low – No selective reporting. | Moderate |

**Supplementary Table 3. Secondary outcomes reported in included studies**

| **Study** | **Outcome** | **Definition (as reported)** | **IONM group (n/N, %)** | **No IONM group (n/N, %)** | **Reporting note** |
| --- | --- | --- | --- | --- | --- |
| **Cofano et al., 2020** | Extent of resection | GTR based on postoperative MRI | Not reported per arm | Not reported per arm | EOR reported for cohort; no stratification by IONM |
| **Cofano et al., 2020** | Length of stay | Mean hospital LOS | Not reported per arm | Not reported per arm | LOS reported only globally |
| **Cofano et al., 2020** | Complications | CSF leak, wound infection, reoperation | Not stratified | Not stratified | Complications reported without IONM grouping |
| **Mirza et al., 2024** | Extent of resection | Surgeon-reported GTR | Not reported per arm | Not reported per arm | GTR discussed, no numerical stratification |
| **Mirza et al., 2024** | Length of stay | Median LOS | Not reported | Not reported | LOS not stratified by monitoring |
| **Mirza et al., 2024** | Complications | Aggregate postoperative complications | Not stratified | Not stratified | Complications reported globally |
| **Harel et al., 2017** | Extent of resection | GTR vs STR | Not reported | Not reported | EOR not reported by monitoring status |
| **Harel et al., 2017** | Complications | New neurological deterioration | Reported | Reported | Neurological outcome used as primary endpoint |
| **Cabañes-Martínez et al., 2024** | Extent of resection | GTR vs STR | Not reported per arm | Not reported per arm | Explicitly stated as similar |
| **Cabañes-Martínez et al., 2024** | Complications | CSF leak, infection, reoperation | Not stratified | Not stratified | Low rates reported, no per-arm counts |

**References:**

1. Azad TD, Pendharkar AV, Pan J, Chen J, Feng AY, Tamura RS, et al. Intraoperative neurophysiological monitoring for intramedullary spinal cord tumor resection: a systematic review and meta-analysis. *Clin Spine Surg*. 2018;31(3):112-119. doi:10.1097/BSD.0000000000000644.
2. Cabañes-Martínez L, Menéndez-García A, Maza-Bustillo M, Morales F, Carrasco-García I, López-González F, et al. Intraoperative neurophysiological monitoring in intradural extramedullary spinal tumor surgery: functional outcomes and prognostic factors. *J Clin Med*. 2024;13(24):7588. doi:10.3390/jcm13247588.
3. Clark AJ, Safaee MM, Oh T, Barber J, Lad SP, Dahdaleh NS, et al. Intraoperative neurophysiological monitoring in contemporary spinal surgery: a systematic review of clinical outcomes and cost-effectiveness. *Neurosurg Focus*. 2020;48(5):E9. doi:10.3171/2020.2.FOCUS2015.
4. Cofano F, Bertero L, Di Perna G, Massaro F, Tardivo V, Ajello M, et al. Intraoperative neurophysiological monitoring and spinal intradural extramedullary tumors: a single-center experience with a focus on long-term functional outcome. *Front Neurol*. 2020;11:598619. doi:10.3389/fneur.2020.598619.
5. Engelhard HH, Villano JL, Porter KR, Stewart AK, Barker FG 2nd, Newton HB. Clinical presentation, histology, and treatment in 430 patients with primary tumors of the spinal cord: results of a multicenter study. *J Neurosurg Spine*. 2010;13(1):67-77. doi:10.3171/2010.3.SPINE09499.
6. Guyatt GH, Oxman AD, Vist GE, Kunz R, Falck-Ytter Y, Alonso-Coello P, Schünemann HJ; GRADE Working Group. GRADE: an emerging consensus on rating quality of evidence and strength of recommendations. *BMJ*. 2008;336(7650):924-926. doi:10.1136/bmj.39489.470347.AD.
7. Harel R, Schleifer D, Appel S, Attia M, Cohen ZR, Knoller N. Spinal intradural extramedullary tumors: the value of intraoperative neurophysiologic monitoring on surgical outcome. *Neurosurg Rev*. 2017;40(4):613-619. doi:10.1007/s10143-017-0815-2.
8. Ishida W, Yokoyama K, Takahashi S, Matsumoto M, Imagama S, Ito Z, et al. Diagnostic and therapeutic values of intraoperative electrophysiological monitoring for intradural extramedullary spinal cord tumors. *J Neurosurg Spine*. 2019;31(5):722-731. doi:10.3171/2019.6.SPINE1978.
9. Kim CH, Chung CK, Jahng TA. Surgical outcome of spinal canal meningiomas: experience with 60 patients. *J Korean Neurosurg Soc*. 2007;42(4):300-304. doi:10.3340/jkns.2007.42.4.300.
10. MacDonald DB, Dong C, Quatrale R, Sala F, Toleikis JR, Kothbauer KF, et al. Recommendations of the International Society of Intraoperative Neurophysiology for intraoperative monitoring of the corticospinal tract during spinal surgery. *Clin Neurophysiol*. 2019;130(1):120-137. doi:10.1016/j.clinph.2018.10.016.
11. Mirza A, Debono B, Cornelis F, Sabatier P, Afathi M, Galanaud D, et al. Impact of intraoperative neurophysiological monitoring on outcomes of intradural extramedullary spinal tumor surgery: a multicenter retrospective cohort study. *Global Spine J*. 2024;14(1):44-53. doi:10.1177/21925682221139822.
12. Sala F, Manganotti P, Tramontano V, Bricolo A, Gerosa M. Monitoring of motor pathways during brain and spinal surgery. *Curr Opin Neurol*. 2007;20(6):686-691. doi:10.1097/WCO.0b013e3282f1ed3e.
13. Scibilia A, Raco A, Miscusi M, Delfini R, Meglio M, Forcato S, et al. The role of intraoperative neurophysiological monitoring for intradural extramedullary spinal tumor surgery. *Neurosurg Focus*. 2016;41(2):E18. doi:10.3171/2016.5.FOCUS16158.
14. Setzer M, Vatter H, Marquardt G, Seifert V, Vrionis FD. Management of spinal meningiomas: surgical results and a review of the literature. *Neurosurg Focus*. 2007;23(4):E14. doi:10.3171/FOC-07/10/E14.
15. Sterne JA, Hernán MA, Reeves BC, Savović J, Berkman ND, Viswanathan M, et al. ROBINS-I: a tool for assessing risk of bias in non-randomised studies of interventions. *BMJ*. 2016;355:i4919. doi:10.1136/bmj.i4919.
16. van der Wal G, Romijn J, Versteeg AL, Jacobs WC, Peul WC, Harhangi BS, et al. The diagnostic value of intraoperative neurophysiological monitoring for early postoperative neurological deficits in spinal surgery: a systematic review. *Clin Neurophysiol Pract*. 2021;6:1-7. doi:10.1016/j.cnp.2020.12.001.
17. Ouzzani M, Hammady H, Fedorowicz Z, Elmagarmid A. Rayyan — a web and mobile app for systematic reviews. *Syst Rev*. 2016;5:210. doi:10.1186/s13643-016-0384-4.
18. Rijs K, Klimek M, Walchenbach R, Noordmans HJ, Vincent AJ. Intraoperative neurophysiological monitoring for the detection of impending neurological injury during resection of spinal tumors: a systematic review and meta-analysis. *World Neurosurg*. 2019;125:498-510.e2. doi:10.1016/j.wneu.2019.01.216.
